# Reviewers in 2024

**DOI:** 10.1098/rsif.2025.0215

**Published:** 2025-05-07

**Authors:** Richard Cogdell

**Affiliations:** ^1^ Editor-in-Chief

I thank all those referees who helped assess submissions to the *Journal of the Royal Society Interface* (volume 21) in 2024. The editorial team very much appreciates the time and dedication given by academics to the peer-review process and recognizes their efforts. Based on previous feedback, we know that most reviewers value recognition of their efforts by the journal and through services, such as the Web of Science Reviewer Recognition Service, which is integrated with *J. R. Soc. Interface*.

To provide an opportunity for recognition, we list the names of all reviewers who provided reports last year (unless they have opted out or not responded). This article is made permanent and citable through its digital object identifier (DOI). We encourage you to quote your contribution in your applications for tenure, grants or other forms of research assessment. Where you have provided it, we have included your ORCID so your contribution can be unambiguously assigned to you.

The team really appreciates all the work our reviewers do for the journal, and we look forward to working with you again in 2025.

Ahmed T

Alexander M

Almeida-Warren K

Amarpuri G

https://orcid.org/0000-0002-2655-5974

Anand M

https://orcid.org/0000-0002-2425-4502

Angin S

Antonioni A

Arrieta Sanagustin J

https://orcid.org/0000-0003-1260-6168

Bai S

https://orcid.org/0000-0002-8746-3096

Banerjee S

https://orcid.org/0000-0001-8000-2556

Bartolucci G

Baskonus HM

https://orcid.org/0000-0003-4085-3625

Bau J

Bauzile B

Bayly PV

https://orcid.org/0000-0003-4303-0704

Behling A-V

https://orcid.org/0000-0003-0844-4143

Bershteyn A

Bhamla S

https://orcid.org/0000-0002-9788-9920

Biegański P

https://orcid.org/0000-0003-2598-6526

Borazjani I

https://orcid.org/0000-0001-7940-3168

Boyer C

https://orcid.org/0000-0002-1101-2045

Broom M

Capraro V

https://orcid.org/0000-0002-0579-0166

Carroll E

https://orcid.org/0009-0001-8981-5024

Cassidy T

Castellano C

https://orcid.org/0000-0002-3773-3801

Cauchemez S

Caudron F

Cecchini D

Chachlaki K

Challita E

Chaw RC

Cheng J

Cheng K

https://orcid.org/0000-0002-4913-2691

Chipman A

Chirarattananon P

https://orcid.org/0000-0003-0142-8394

Chizh A

Chu E

Ciarchi M

Clark C

https://orcid.org/0000-0001-7943-9291

Coffin JM

Conway D

Correa SB

Cowan N

Cretu A-M

Cylke A

De Meijere G

https://orcid.org/0000-0002-2112-2256

De-Oliveira-E-Silva M

https://orcid.org/0009-0007-7140-5004

DeFelice N

Delli V

https://orcid.org/0000-0001-9137-8735

Di Stefano A

Di Z

Diambra L

https://orcid.org/0000-0001-8052-4880

Dobrovolny H

https://orcid.org/0000-0003-3592-6770

Dona M

Doyon J

https://orcid.org/0000-0002-3904-7207

Drake J

https://orcid.org/0000-0003-4646-1235

Drevensek S

Dubljevic V

Duennwald ML

Dunn I

Durka P

D’Alba L

Eguchi S

Elbaum R

https://orcid.org/0000-0003-4417-3811

Erinjery J

Everitt R

Fages F

Fellermann H

Fillingham P

https://orcid.org/0000-0002-8139-7331

Finnilä M

Fintzi J

Fleischmann S

Flores J

Flores K

https://orcid.org/0000-0002-4000-6971

Forbes P

Fosbury R

https://orcid.org/0000-0001-9975-8003

Francois P

French D

Fu H

https://orcid.org/0009-0006-5419-2388

Garuma GF

Gaurav K

https://orcid.org/0000-0001-8119-297X

Ghanbari A

Gill A

https://orcid.org/0000-0001-5019-6054

Gill J

https://orcid.org/0000-0002-4115-8045

Glasser J

https://orcid.org/0000-0003-3451-5849

Goh K

Gonzalez-Parra G

Goodin D

Gurupur V

Gómez-Benito MJ

Han TA

https://orcid.org/0000-0002-3095-7714

Heinrich M

https://orcid.org/0009-0009-4267-7195

Hemery M

Hickson R

https://orcid.org/0000-0001-6453-7745

Hinton A

Hipwell C

Hof A

Houssiau F

https://orcid.org/0000-0001-7412-4389

Ibrahimli R

Jagota A

https://orcid.org/0000-0002-0779-0880

Ji X

Jiang J

Jung S

https://orcid.org/0000-0002-1420-7921

Katti P

Kaur J

Keddie J

Kelly L

https://orcid.org/0000-0002-9736-0517

Kenaley C

https://orcid.org/0000-0003-3832-7001

Kessel A

Kheiri S

https://orcid.org/0000-0002-9934-4297

Kiemel T

https://orcid.org/0000-0001-6970-7272

King A

https://orcid.org/0000-0001-6159-3207

Koelewijn A

https://orcid.org/0000-0002-1867-0374

Kolomenskiy D

Korin N

https://orcid.org/0000-0001-7244-889X

Korolev K

Krasnyakov I

Kröger R

Laeverenz-Schlogelhofer H

https://orcid.org/0000-0003-2958-5711

Lange A

Leeuwis M

https://orcid.org/0009-0009-8728-0589

Lenain L

Lipiecki A

https://orcid.org/0000-0003-1118-0388

Liu H

https://orcid.org/0000-0002-8687-3237

Lunau K

https://orcid.org/0000-0001-5184-4201

Lushi E

MacIntosh B

Marathe A

Marcon L

https://orcid.org/0000-0003-0957-9170

Martin R

Matthew R

https://orcid.org/0000-0003-2373-4384

Mazzucco N

https://orcid.org/0000-0002-9315-3625

Mcgettigan R

https://orcid.org/0009-0002-8270-8848

Mizumoto N

https://orcid.org/0000-0002-6731-8684

Morrill A

Moulder R

Mueller J

https://orcid.org/0000-0002-4808-2223

Muller E

https://orcid.org/0009-0007-0924-5898

Musotto G

Nagy M

https://orcid.org/0000-0001-8817-087X

Narla A

https://orcid.org/0000-0002-9328-8271

Neikirk K

Ngonghala C

https://orcid.org/0000-0002-8441-9495

Nishikawa S

https://orcid.org/0000-0003-2858-2164

O'Riordan C

https://orcid.org/0000-0003-0449-8224

Ostinato M

Ozkan-Aydin Y

Pajic-Lijakovic I

https://orcid.org/0000-0001-9663-6916

Parag K

https://orcid.org/0000-0002-7806-3605

Park J

Parkin C

https://orcid.org/0000-0002-2765-0594

Patten M

https://orcid.org/0000-0001-9689-2645

Peaucelle A

Pedro L

https://orcid.org/0000-0001-5339-251X

Perelman S

Pires A

https://orcid.org/0000-0002-0648-5702

Raman K

https://orcid.org/0000-0002-9311-7093

Rathore J

Reluga T

Rist M

Rocklöv J

Romanelli E

Rose JBR

Rubolini D

Ruffo G

Rulands S

Rumpler E

https://orcid.org/0000-0003-2223-2681

Sabetian A

Sadhu RK

Sailash Singh M

https://orcid.org/0000-0003-0503-1693

Saldaña F

Santos FP

https://orcid.org/0000-0002-2310-6444

Sheppard A

Shivkumar VS

https://orcid.org/0000-0001-6094-3041

Singh P

Skandalis D

https://orcid.org/0000-0002-5126-9582

Smith J

Spacapan M

Specht I

https://orcid.org/0000-0003-2834-8191

Stocker R

Sun Y

Sznajd-Weron K

Tal O

Tasse A

Taylor B

https://orcid.org/0000-0002-0604-769X

Tepole AB

Thomson D

Timmer J

Tischler D

https://orcid.org/0000-0002-6288-2403

Toaquiza Tubon J

https://orcid.org/0000-0001-5778-1109

Trapman P

Tsanou B

Tu Y

https://orcid.org/0000-0002-4589-981X

Umeh B

Unger, J

https://orcid.org/0000-0003-4832-5675

Usmani O

Vallejo-Marin M

https://orcid.org/0000-0002-5663-8025

van de Leemput I

van der Zee J

https://orcid.org/0000-0003-3673-173X?lang=en

Van Dieen J

https://orcid.org/0000-0002-7719-5585

Van Schaik P

Velasco Hernández J

Veloz T

https://orcid.org/0000-0003-3014-8340

Venturi V

Verma S

Vlasceanu GM

https://orcid.org/0000-0002-9876-6180

Wallace D

Wan G

https://orcid.org/0000-0002-9479-0861

Wan K

https://orcid.org/0000-0002-0291-328X

Wang S

Ward M

Warrell J

https://orcid.org/0000-0002-1323-4602

West J

https://orcid.org/0000-0001-9579-4664

Wolf S

https://orcid.org/0000-0002-3747-8097

Woodrow C

Wu W

https://orcid.org/0000-0001-5380-7746

Yang S

Yunus AO

Zakaria M

https://orcid.org/0000-0002-5617-8524

Zhang J

https://orcid.org/0000-0001-8809-3405

